# Wasting and Underweight in Northern African Children: Findings from Multiple-Indicator Cluster Surveys, 2014–2018

**DOI:** 10.3390/nu15143207

**Published:** 2023-07-19

**Authors:** Nagwa Farag Elmighrabi, Catharine A. K. Fleming, Kingsley E. Agho

**Affiliations:** 1 School of Health Sciences, Western Sydney University, Campbelltown Campus, Locked Bag 1797, Penrith, NSW 2571, Australia; catharine.fleming@westernsydney.edu.au (C.A.K.F.); k.agho@westernsydney.edu.au (K.E.A.); 2 Organization of People of Determination and Sustainable Development, Benghazi, Libya; 3 Department of Nutrition, Faculty of Public Health, University of Benghazi, Benghazi 1038, Libya; 4 Translational Health Research Institute (THRI), School of Medicine, Western Sydney University, Penrith, NSW 2750, Australia; 5 Faculty of Health Sciences, University of Johannesburg, Johannesburg 2094, South Africa

**Keywords:** undernutrition, predictors, childhood, Algeria, Egypt, Sudan, Tunisia

## Abstract

Northern Africa faces multiple severe detrimental factors that impact child nutrition. This study aimed to identify the predictors for wasting and underweight in children aged 0–59 months in Northern Africa. We analysed pooled cross-sectional data from multiple-indicator cluster surveys conducted in four countries (Algeria, Egypt, Sudan, and Tunisia) involving 37,816 children aged 0–59 months. A logistic regression analysis was used, considering clustering and sampling weights, to identify factors associated with wasting and underweight among children aged 0–23, 24–59, and 0–59 months. Among children aged 0–59 months, the overall prevalence was 7.2% (95% CI: 6.8–7.5) for wasting and 12.1% (95% CI:11.7–12.5) for underweight. Sudan and Algeria had the highest rates of wasting, while Sudan and Egypt had the highest rates of underweight. Multiple regression analyses indicate that factors associated with wasting and being underweight include child age, country, rural residency, poor wealth index, being male, birth order, maternal education, body mass index, media use, lack of diverse foods, longer duration of breastfeeding, perceived small baby size, and diarrhoea. These findings highlight the importance of implementing targeted health and nutrition initiatives, such as maternal education, family planning, and community engagement. Priority should be given to children from underprivileged areas who lack proper dietary variety.

## 1. Introduction

The consequences of undernutrition (stunting, wasting, and underweight) are severe, contributing to 3.1 million deaths annually. Wasted children (0–5 years) are significantly more likely to die than healthy children, with an 11-fold increased risk [1]. Wasting (acute malnutrition) hinders growth, impairs cognitive development, and elevates the risk of non-communicable diseases in adulthood. Along with this, wasting weakens a child’s immune system, resulting in a young child becoming more susceptible to infectious diseases and heightening the risk of death [1]. The physical health of children deteriorates rapidly when they experience weight loss because of illness or inadequate nourishment [2].

Undernutrition encompasses stunting, wasting, underweight, and micronutrient deficiencies; for this study, we focused specifically on wasting and underweight among children under five in Northern Africa due to their prevalence in the region and to protect the most vulnerable children from food and nutrition crisis [3,4]. This remains a significant global challenge, with 45 million children suffering from wasting (too thin for height) and 462 million from underweight (too thin for age) [5]. In particular, North African countries, including Egypt, Libya, Sudan, Algeria, Morocco, Tunisia, and Western Sahara, face a high burden of undernutrition among children. Despite global efforts to address this issue, the prevalence of wasting and underweight in North Africa continues to rise [5].

Recent data from a systematic and meta-analysis study show that the overall pooled prevalence of wasting among children under five in North Africa is approximately 7.9%, exceeding both the global average of 6.7% and the value observed in West Africa [3,6]. Similarly, the prevalence of underweight among children under five in North Africa is approximately 12.9%, surpassing the global average of 12.6% [3,7]. These statistics highlight the increasing burdens of undernutrition in the region, signalling a critical need for action.

Multiple interrelated factors contribute to undernutrition, including socioeconomic factors, a child’s environment, food insecurity, economic hardship, armed conflicts, global warming, immigration, poverty, inequality, and the spread of communicable diseases. These are key determinants of malnutrition in low- to middle-income countries [8]. Children in North Africa face these same challenges, which exacerbate immediate factors such as maternal health, dietary intake, and diseases [9,10,11,12].

Addressing child acute malnutrition is a priority in global efforts to combat the food and nutrition crisis, as it leads to significant mortality rates in countries affected by underlying factors [13]. The UNICEF’s Acceleration Plan for 2022–2023 emphasises early prevention, detection, and treatment of wasting in the most vulnerable countries [13]. However, access to treatment remains critically low, necessitating the development of scalable solutions for both treatment and prevention [13]. Unfortunately, there is a lack of up-to-date monitoring and assessment programs in most North African countries, which may contribute to the severity of the problem [3,14]. 

Furthermore, it is important to note that most countries in Northern Africa are not currently the focus of global action plans addressing undernutrition. While Sudan is included in the Global Action Plan for Child Wasting among fourteen other countries, countries such as Libya and Egypt have not received sufficient attention [4]. Libya, for instance, continues to face challenges such as political instability, high food insecurity, and unmanaged climate change, which contribute to the prevalence of wasting and underweight among children. Similarly, Egypt struggles with a high level of poverty, further exacerbating the undernutrition situation in the country [15,16].

Recognising the importance of investing in data, monitoring, and evaluation for nutrition, national and international strategies and policies have emphasised the need for timely and high-quality nutrition information [4,17]. These data are crucial for guiding program actions, allocating resources effectively, and promoting accountability [18,19]. Therefore, this study aims to investigate the determinants of wasting and underweight among children under five in four North African countries: Egypt, Sudan, Algeria, and Tunisia. By providing updated information, this study seeks to contribute to efforts aimed at addressing and mitigating the impact of undernutrition in the region.

## 2. Materials and Methods

Multiple-indicator cluster surveys (MICSs) are a validated data collection tool used to gather essential information on various factors of maternal and child health, education, child mortality, child protection, HIV/AIDS, and water and sanitation [20]. These data play a vital role in advocacy and policy development. Additionally, MICS findings are crucial for countries to assess their progress towards achieving sustainable development goals. In many countries, MICSs serve as the primary source of reliable population-based data on children and women and are a significant component of national statistical plans [20]. These surveys were compatible with the aims of this study given the standardised nature of the data collection methods and instruments.

To collect MICS data, surveyors administer questionnaires to different groups, including women and men aged 15 to 49, mothers or caregivers of children under 5, and randomly selected children aged 5–17. MICSs focus on coverage indicators and investigate knowledge, attitudes, and behaviours related to various topics. This comprehensive approach allows analysts to gain insights into the factors influencing the lives of women and children. The data are disaggregated by age, gender, education, wealth, location, ethnicity, and other categories to identify disparities and understand which groups are being left behind [21]. 

For our study, we incorporated the most recent rounds of publicly accessible MICS data sets (2014–2018) from four countries in Northern Africa: Egypt (2014), Sudan (2014), Algeria (2018), and Tunisia (2017). In total, the survey included 37,816 children aged 0 to 59 months, with 16,355 from Algeria, 5053 from Egypt, 12,793 from Sudan, and 3615 from Tunisia. We utilised the anthropometry module from the questionnaire for children under five; the maternal, education, and household characteristics modules from the household and women questionnaires; and data on child anthropometric variables, age, gender, maternal education, household characteristics, and urbanity.

For a comprehensive list of indicators and additional information on MICS topics, please visit https://mics.unicef.org/ (accessed on 21 June 2023).

### 2.1. Dependent Variables

The weight-for-height index is a metric that gauges an individual’s body mass relative to their height and provides insights into their present nutritional status. This index was derived using growth standards outlined by the World Health Organization (WHO) in 2006 [22]. These standards were established based on data collected during the WHO Multicentre Growth Reference Study and are expressed in standard deviation units from the study’s median. When children exhibit weight-for-height Z-scores that fall below minus two standard deviations (−2 SDs) from the median of the reference population, they are categorised as wasted or acutely malnourished [22]. Similarly, the underweight index considers both acute malnutrition and chronic malnutrition (stunting) by integrating height-for-age and weight-for-height measurements. Children whose weight-for-age is below minus two standard deviations (−2 SDs) from the median of the WHO Multicentre Growth Reference Study are classified as underweight. The survey presented the indicators for wasting and underweight as a dependent dichotomous variable, where category 0 referred to individuals who were not wasted or underweight (with Z-scores > −2 SDs) and category 1 encompassed those who were wasted or underweight (with Z-scores < −2 SDs) [22].

### 2.2. Independent Variables

**Figure 1:**
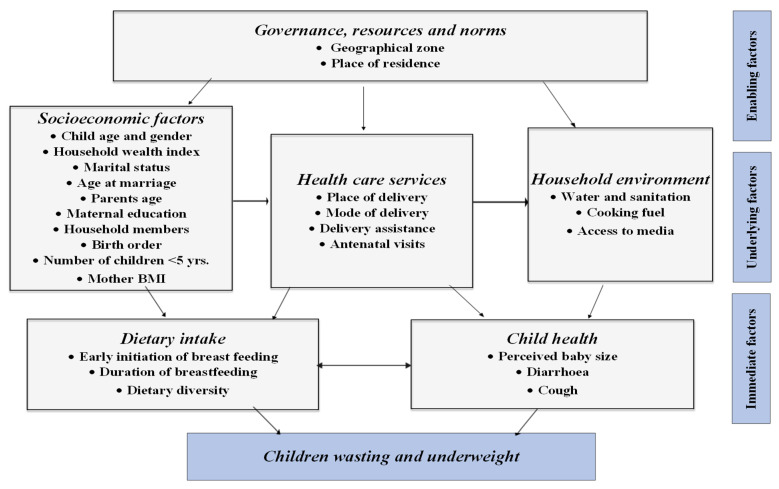
Adopted study factors associated with wasting and underweight among children under five in Northern Africa based on the UNICEF conceptual framework for the determinants of nutritional status for maternal child’s health.

The classification of factors investigated in our study was based on modifying UNICEF’s Conceptual Framework on the Determinants of Maternal and Child Nutrition, 2020, which identifies three key determinants for child and maternal nutritional health: enabling, underlying, and immediate determinants [23]. This modification drew upon the study conducted by Zhihui et al. in 2020 [24], which specifically investigated 35 countries classified as low-middle-income countries. The confounding variables were subdivided into three groups: enabling factors, including geographical zone (country) and place of residence (urban/rural); underlying factors, including the pooled household wealth index, child age and gender, parents’ age, maternal education and nutritional health, household members, children under 5, birth order, health care services use, water and sanitation, cooking fuels, and access to media; and immediate factors, including dietary intake, early initiation of breastfeeding, duration of breastfeeding, perceived baby size, and child illness (diarrhoea and cough in the last two weeks). (Figure 1).

The classification of independent variables is provided in detail in Table 1. However, for enhanced clarity, certain variables were further identified as follows: In the case of the surveys conducted in Egypt, Sudan, Algeria, and Tunisia, the pooled household wealth index was calculated using the wealth score (wscore) variable. The “wscore” for each country was computed using the statistical method of principal components analysis (PCA). The PCA method was carried out using the information obtained from the ownership of consumer goods, dwelling characteristics, water and sanitation, and other assets and durables that are related to the household’s wealth index [25]. This household wealth index was divided into five categories and each household was assigned to one of these wealth index categories. The bottom 20% of the household wealth index was arbitrarily referred to as the poorest households, and the top 20% was referred to as the richest households. Cleaned cooking fuels were identified as solar, electric, gas, natural gas, liquid petroleum gas, and alcohol fuels [26]. Improved toilet facilities were identified as flush or pour-flush to a piped sewer system, septic tank pit latrines, ventilated improved pit latrines, or pit latrines with a slab or composting toilets [26]. Protected sources of drinking water were defined as household connections, public standpipes, boreholes, protected dug wells, protected springs, and rainwater collection [27]. Dietary diversity was identified based on a score ranging from (0 to 8): breast milk; grains, roots, and tubers; legumes and nuts; dairy products (milk, yogurt, and cheese); flesh foods (meat, fish, poultry, liver, and organ meats); egg; vitamin A rich fruits and vegetables; and other fruits and vegetables. The dietary diversity was categorised as 5 or more food groups or less than 5 food groups [28]. Perceived baby size was classified as small = less than 2500 g; average = 2500–4000 g; and large = >4000 g [29].

### 2.3. Statistical Analysis

**Figure 2:**
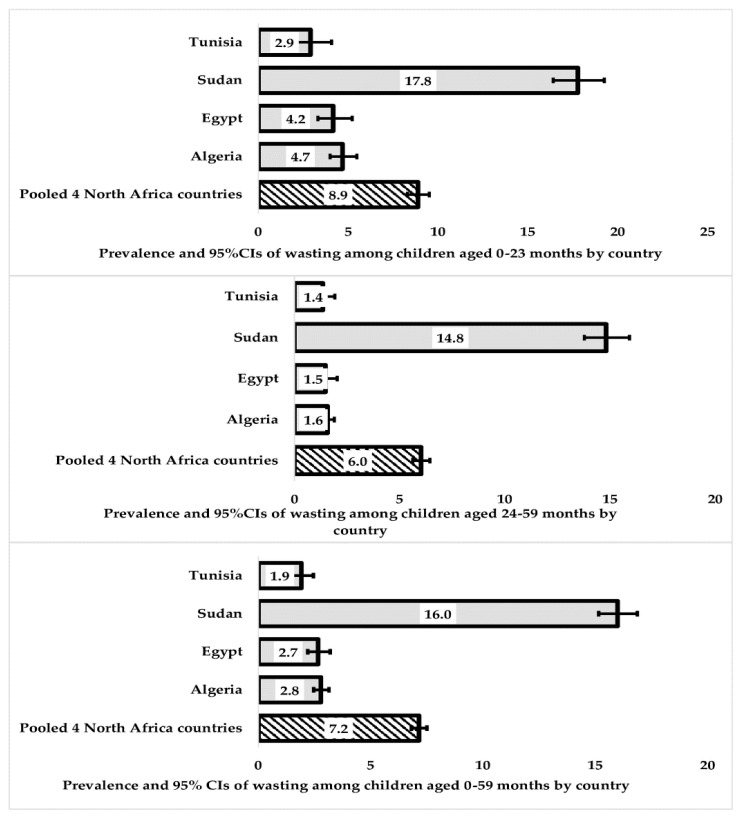
Prevalence and 95% CIs of wasting among children (0–23, 24–59, and 0–59 months) in four Northern African countries.

**Figure 3:**
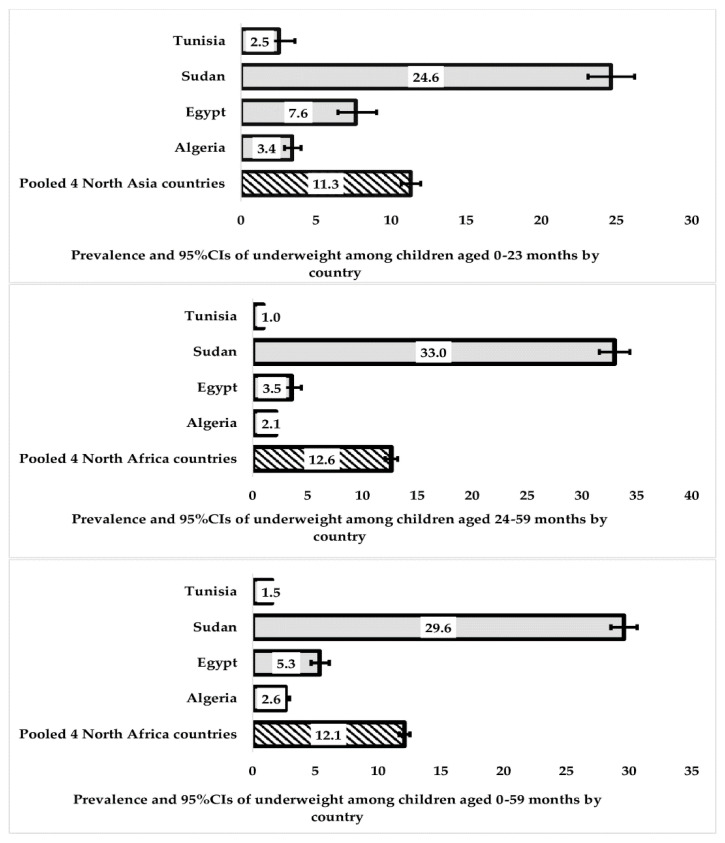
Prevalence and 95% CIs of underweight among children (0–23, 24–59, and 0–59 months) in four Northern African countries.

In our initial analysis, we used country-specific weights in the Taylor series linearisation method to estimate 95% confidence intervals around the prevalence of wasting and underweight in each country, as described in Figure 2 and Figure 3. In order to ensure accurate results, we took several steps in our statistical analysis. First, we adjusted the sampling weights by dividing each country’s sampling weights by the total sum of weights for all four countries. This allowed us to create a sampling weight called “population weights”. Additionally, we created a unique country-specific cluster to account for the variation in clustering for each country. This was necessary because a large population in one country (such as Egypt, with over 103 million people in 2018) could offset countries with smaller populations (such as Tunisia, with about 11 million people in 2018) [30].

To account for population weights and the country-specific cluster sampling design, the “SVY” commands in Stata version 17.0 (Stata Corp, College Station, TX, USA) were utilised for data analysis. Firstly, a bivariate binary logistic regression analysis, which adjusted for population weights and country-specific clustering, was performed to determine the unadjusted odds ratio of wasting and underweight among children aged 0–23 months, 24–59 months, and 0–59 months. Secondly, a multiple logistic regression, which adjusted for population weights and country-specific clustering, was used in a manual backward elimination manner to identify the factors significantly associated with wasting and underweight among children under five.

In these analyses, a staged modelling technique was used for the multivariable analysis, in which each level in the UNICEF conceptual framework was entered progressively into the model to assess their relationship with this study’s outcomes [31]. The enabling factors were first entered into the multivariable model to determine their association with this study’s outcomes. A manual stepwise backwards elimination process was conducted, and only the variables that were significantly related to this study’s outcomes at *p* < 0.05 were retained in the model (step 1). Next, the underlying factors were added to step 1, and only those with *p*-values < 0.05 were retained (step 2) after a manual backwards elimination process. Lastly, the immediate factors, such as dietary intake and child health factors, were added to step 2, and only the variables with a statistical significance of *p* < 0.05 were retained in the final model.

All variables with a statistical significance of *p* < 0.05 were retained in the final model. The outcome variables considered were (i) wasting at 0–23 months, (ii) wasting at 24–59 months, (iii) wasting at 0–59 months, (iv) underweight at 0–23 months, (v) underweight at 24–59 months, and (vi) underweight at 0–59 months. A multiple logistic regression model was used to determine the adjusted odds ratio.

## 3. Results

### 3.1. Sociodemographic Characteristics of the Study Population

A total of 37,816 children aged 0–59 months were included in this study to determine their nutritional health. The data used for the analysis came from all four MICS surveys, with 43.25% of the children from Algeria, 13.36% from Egypt, 33.83% from Sudan, and 9.56% from Tunisia.

The sample consisted of more children in the age range of 24–59 months, with a proportion of 2:3 compared to children aged 0–23 months. The sample demonstrated an approximately even distribution among males and females and across different places of residence and family financial statuses.

In terms of household composition, 66.09% of the children lived in households with 5–10 people. Additionally, 54.25% of the families had two or more children under the age of five, while 10.78% had four or more siblings. The majority of mothers examined were aged 20–34, whereas the majority of fathers examined were aged 35–44. Furthermore, approximately 24.75% of women were married before turning 18.

The educational background of the mothers revealed that 64.92% had either no education or a low level of education. Moreover, 84.88% of women had a body mass index (BMI) of 18.5 or lower. While a large number of women reported watching TV, fewer women reported listening to the radio. For more detailed information, refer to Table 2.

Regarding healthcare utilisation, 28.21% of the women gave birth at home; 21.2% had between one and three antenatal clinic visits; and 17.86% of the women did not see a doctor during their pregnancy. However, 82.79% received professional assistance during childbirth.

Concerning child health, dietary consumption, and feeding practices, 23.72% of the children were born small (<2500 g), with a weight below normal. Approximately seven out of ten children lacked access to diverse foods, and nearly half began breastfeeding after an hour of birth. Furthermore, 16.35% of the studied children had experienced diarrhoea in the last two weeks, and 28.52% had a cough.

The environmental conditions where the child lived were a cause for concern. Around 44.38% of families had unimproved toilet facilities, 24.8% needed access to clean water, and approximately 21.13% of families used unclean fuel for cooking (see Table 2 for more information).

### 3.2. Prevalence of Wasting and Underweight in Northern Africa

Figure 2 displays the prevalence of wasting (acute undernutrition), specifically weight-for-height z-scores according to the 2006 WHO standards, among children aged 0–23 months, 24–59 months, and 0–59 months in Northern Africa. The prevalence of wasting, a form of acute undernutrition, among children aged 0–59 months was 7.2% (95% CI: 6.82, 7.51), with the highest prevalence observed in Sudan at 16.0% (95% CI: 15.16, 16.88). Wasting was more common among younger children aged 0–23 months, with a prevalence of 8.9% (95% CI: 8.29, 9.52), while Algeria and Egypt had relatively low-to-moderate prevalence rates of 4.7% (95% CI: 4.00, 5.48) and 4.2% (95% CI: 3.31, 5.23), respectively.

Figure 3 provides an overview of the combined prevalence of underweight, as determined using weight-for-age z-scores in relation to the 2006 WHO standards, among children aged 0–23 months, 24–59 months, and 0–59 months in the Northern African region and each of the four investigated countries. In children aged 0–59 months, the overall pooled prevalence of underweight was higher than that of wasting, at 12.1% (95% CI: 11.68, 12.54). Among specific age groups, underweight was less prevalent among children aged 0–23 months, with a prevalence of 11.3% (95% CI: 10.67, 11.97), while it was highest among children aged 24–59 months at 12.6% (95% CI: 12.07, 13.21). 

It is worth noting that while underweight was more prevalent among older children in Sudan, with a prevalence of 33% (95% CI: 31.6, 34.38), it was more common among younger age groups in other countries. For more details on the prevalence of wasting and underweight across different factors, refer to Table S1.

### 3.3. Indicators for Wasting in Northern Africa

Table 3 presents the variables significantly associated with the presence of wasting among children aged 0–23 months, 0–24 months, and 0–59 months in Northern Africa. Geographical location emerged as the primary influential factor affecting the prevalence of wasting among all children under the age of five in the region. Specifically, children from Sudan and Algeria demonstrated a higher likelihood of experiencing wasting, while children from Tunisia exhibited a lower probability of being affected. Moreover, children who had limited access to diverse food groups had higher odds of being wasted compared to children with better access to a diverse range of foods, regardless of their ages. However, when considering only younger children in the region, being in a higher birth order category was found to be associated with a higher probability of experiencing wasting. Among older children, the analysis demonstrated that females had a lower likelihood of being wasted than males, that wasting was more prevalent in households with lower wealth levels, that children with older fathers had a decreased risk of wasting, and that experiencing diarrhoea increased the likelihood of wasting compared to those without diarrhoea.

### 3.4. Indicators for Underweight in Northern Africa

Table 4 displays the number of variables that showed a significant association with the prevalence of underweight among children in two age groups: 0–23 months and 0–59 months, specifically in Northern Africa. Regardless of age, certain factors were consistently linked to underweight in this region. Similar to the wasting indicators, children living in specific geographical zones within Northern Africa displayed a higher likelihood of experiencing underweight. Notably, Sudan and Egypt were countries where children had a greater prevalence of underweight compared to Algeria or Tunisia. Children residing in rural areas were also more vulnerable to underweight. Another critical factor associated with being underweight was the household’s wealth index. Children from households with lower wealth indices were more prone to experiencing underweight. Additionally, our study indicated that children who had suffered from diarrhoea within the previous two weeks were more susceptible to being underweight. 

In terms of age groups, the multivariable analysis indicated that children aged 18–23 months and 30–35 months were more likely to be underweight compared to other age groups. Analysing age-specific associations, this study identified additional factors that influenced underweight among children. In younger age groups, factors such as a lack of diverse foods, prolonged breastfeeding for more than 12 months, and being born with either a small or large size were associated with being underweight. Female children, particularly in the young age groups of < 23 months, were found to be less likely to be underweight compared to males.

Conversely, certain factors were more strongly linked to older age groups. Factors such as low maternal education level, low maternal body mass index (BMI), and lack of media exposure, specifically, not listening to the radio or not watching TV, were identified as statistically significant factors associated with underweight among children in Northern Africa. 

## 4. Discussion

The prevalence of wasting and underweight in Northern Africa is a matter of great concern, as indicated in the current results that combine national representative data from Algeria, Egypt, Sudan, and Tunisia. Sudan faces a higher-than-average overall prevalence of undernutrition, with 7.2% of children experiencing wasting, and 12.1% being underweight. To better understand the factors contributing to undernutrition, our study examined various domains based on UNICEF. The prevalence of wasting was influenced by geographical location and lack of diverse food across different age groups. Among young age groups, birth order played a significant role in wasting. Older age groups were more likely to experience wasting if they had an older father or recent episodes of diarrhoea. Interestingly, being female acted as a protective factor against wasting in older individuals. Various factors contributed to underweight in Northern Africa, including geographical location, rural residence, lower household wealth indices, experiencing diarrhoea, specific age groups, and lack of access to diverse foods, which affected all age groups. Similarly, being female was a protective factor against underweight. Among older age groups, low maternal BMI, limited access to media, and low maternal education levels were more prevalent. On the other hand, prolonged breastfeeding (>12 months) and perceived baby size were more likely among young age groups associated with underweight.

Our study results demonstrate how a child’s geographical location is the most correlated factor associated with wasting and underweight among all age groups. Governance and potential resources play a highly significant role in shaping the nutritional health of children in Northern Africa. The quantity and quality of actual resources, human and economic organisation, environment, and national policies have a crucial impact on maternal health, well-being, and the growth of children [32,33,34]. However, North African countries face several challenges that contribute to the presence of and variation in undernutrition across different geographical areas. These challenges include political instability, conflicts, poverty, inequality, climate change, food insecurity, and the presence of infectious diseases [8,11,16,35,36]. Conflicts, for instance, lead to a decline in public services, higher food and fuel prices, and loss of livelihoods, ultimately reducing food access and posing significant protection challenges for children and families [16,37]. Numerous nutritional surveys have documented the elevated occurrence of malnutrition in countries affected by conflicts [10,37,38]. The effects of climate change exacerbate the situation by disrupting agriculture, causing crop failures, and decreasing food availability, which in turn affects the diversity of nutrients in people’s diets [39]. Moreover, poverty and inequality intensify these challenges by limiting the availability and accessibility of nutritious diverse food, access to healthcare services, and educational opportunities [40]. Poverty and unemployment rates are already high in Algeria, Tunisia, and Libya and are unevenly distributed across age, gender, and geography. These inequalities are major obstacles to the global goal of ending global poverty by 2030, with Africa representing the third most unequal region after Latin America and the Middle East [11]. The consequences of food insecurity and a lack of dietary diversity are particularly severe for child and maternal health, leading to impaired growth and development [41]. 

Our study supports the strong link between wasting and underweight among children in Northern Africa and the lack of diverse foods in the area. The insufficient intake of macronutrients (proteins, carbohydrates, and fats) and essential micronutrients (vitamins and minerals) can lead to malnutrition [42]. Poor dietary diversity and lack of nutrient-rich foods contribute to the development of undernutrition. Our findings show that extended breastfeeding for more than 12 months was significantly linked to wasting in Northern African children. Similarly, two studies in West Africa and Nigeria found that the phase encompassing birth to 6 months, during exclusive breastfeeding, and from 6 to 23 months represents a critical period during which children are particularly susceptible to undernutrition [43,44]. If breastfeeding is prolonged without appropriate intake of diverse nutritious foods that meet the nutritional requirements of the growing child, it can lead to undernutrition and frequent illness [45].

Our study also reported a link between wasting in older children and underweight among all age groups and the presence of diarrhoea in the region. This complex and interconnected link is between child health (specifically, diarrhoea), dietary intake, and child underweight. Diarrhoea is a common childhood illness that can have a detrimental impact on nutritional status [46]. Persistent or recurrent diarrhoea can lead to nutrient loss, reduced absorption, and increased metabolic demands, ultimately contributing to wasting and underweight. Diarrhoea often occurs as a result of poor hygiene practices, contaminated water, inadequate sanitation, and limited access to healthcare [47]. It is important to note that these factors often operate in a vicious cycle. Poor dietary intake can compromise a child’s immune system, making them more susceptible to infections such as diarrhoea [35,46,47]. 

A child’s birth weight has an impact on their nutrition and overall health, and our study found a strong association between low birth weight and underweight. Children with low birth weight are more vulnerable to infections, including diarrhoeal and lower respiratory infections, which contribute to undernutrition. Low birth weight is also connected to inadequate maternal nutrition, low maternal BMI, limited maternal education, and reduced access to antenatal care [48,49,50]. Our findings also indicate a higher likelihood of underweight children among mothers with a low BMI, in comparison to those with a normal or high BMI. This aligns with previous national and regional studies conducted in various locations, suggesting a consistent pattern across different populations [9,51]. Maternal malnutrition can have profound consequences for both mothers and their children. Insufficient nutrient intake during pregnancy can hinder the optimal growth and development of the foetus, leading to a higher risk of underweight children. Additionally, maternal malnutrition can negatively impact the health and well-being of the mothers themselves [52]. It is important to address these factors to improve maternal and child health outcomes. Enhancing maternal nutrition, promoting adequate antenatal care, and addressing socioeconomic factors can contribute to better birth outcomes and reduce the prevalence of underweight children.

Furthermore, our findings indicate a notable association between underweight across various age groups and certain factors, including living in rural areas and having a poor wealth index. It is worth noting that older children from families with a lower wealth index exhibited a higher vulnerability to wasting compared to younger children.

Our results are consistent with two current systematic reviews conducted in both Northern and Sub-Saharan Africa [3,32]. Living in rural areas and having a low maternal education level can significantly impact child health. Limited access to healthcare services in rural areas, including prenatal care and child healthcare, can lead to delays in receiving necessary medical attention, increasing risks for both the mother and child [53]. The health of children in rural areas is further compromised by challenges such as inadequate sanitation, limited availability of nutrient-dense foods, reduced educational opportunities, and poor infrastructure. Families with a low wealth index often struggle to access essential resources, including healthcare, due to limited financial means. This can hinder their ability to provide their children with nutritious food, proper healthcare, and a safe living environment, resulting in negative health outcomes [54]. 

Our results highlight an important aspect of maternal education and its impact on the occurrence of underweight among young children in Northern Africa. Indeed, maternal education level plays a crucial role in several key areas related to child health and development. Mothers with higher education levels tend to have a better understanding of proper nutrition and the importance of early feeding practices. They are more likely to be aware of the benefits of exclusive breastfeeding during the first six months of a child’s life. Additionally, they understand the significance of the timing and frequency of complementary feeding, as well as the importance of diverse food intake. This knowledge empowers them to make informed decisions about their child’s nutrition and overall health. Furthermore, maternal education is closely linked to improved hygiene practices. Educated mothers are more likely to have knowledge about hygiene and sanitation measures that are essential for preventing disease and promoting good health. Mothers with higher education levels are more likely to be aware of available healthcare services and understand the importance of regular check-ups and preventive care for their children. In contrast, mothers with lower education levels may lack knowledge about healthcare practices and may not be aware of the importance of timely medical intervention [54]. Limited education can result in difficulties finding stable employment and lower income potential for parents. Families may have to prioritise other basic needs over seeking of healthcare expenses, potentially resulting in delayed or inadequate healthcare. This lack of access to healthcare services can compromise preventive care, such as vaccinations and regular check-ups, putting children at a higher risk of preventable illnesses and delayed diagnoses of health conditions. [32,34,50,53,55,56]. 

Child age and gender are other important factors that need to be considered when investigating child undernutrition in Northern Africa. Notably, our findings reveal that younger children bear a disproportionately higher burden of wasting, while children aged 24–59 months face an elevated risk of being underweight. Our results are consistent with a number of studies [32,33,56,57]. The nutritional status of mothers significantly impacts the development of their children. Younger children, particularly those under the age of two, are more susceptible to acute undernutrition. This vulnerability can be attributed to factors such as inadequate breastfeeding practices, poor complementary feeding, limited access to nutritious food, and increased susceptibility to infections [44]. Children between the ages of 24 and 59 months (2–5 years old) are more at risk of being underweight (either acute or chronic). This age group is often transitioning from exclusive breastfeeding to family foods, and their nutritional needs increase during this period. Insufficient dietary intake, inadequate nutrient-rich foods, and poor feeding practices can contribute to underweight among these children.

In our study, we found that being a girl was a protective factor for younger ages regarding undernutrition. In the case of younger girls, biological socio-cultural factors may come into play. Research indicates that females tend to have a favourable outcome compared to males, especially during the perinatal period following preterm birth [58]. This suggests a survival advantage for females, as evidenced by their longer lifespan compared to males under challenging circumstances, such as famines and epidemics [59]. For instance, during the famine in Holland in World War II, there was a more pronounced decrease in male births than female births, indicating a higher rate of in utero loss among males [60]. The underlying biological mechanisms that account for these sex differences are still not completely understood and require further exploration [59]. Cultural norms and practices within certain communities or regions might also prioritise the health and well-being of younger girls, leading to better nutrition and care for them compared to boys or older girls. This cultural preference may manifest as increased attention, resources, and investment in the early years of a girl’s life, which can contribute to improved nutritional outcomes [61]. It is important to note that cultural reasons for the protective effect of being a girl at younger ages are context-specific and can vary across different communities and regions. Further research and an understanding of the specific cultural dynamics at play can provide insights into why this protective effect exists and how it can be harnessed to address undernutrition more effectively. 

Our study results regarding underweight also revealed a strong association between childbirth order and father’s age and wasting, listening to the radio, and watching television. Families with a high number of members may experience resource strain, leading to inadequate nutrition, overcrowded living conditions, and limited access to healthcare services, all of which can negatively impact mother and child health [62]. These results are consistent with a number of previous studies [63,64,65]. Having an older father is considered a protective factor against underweight in younger children in Northern Africa, likely due to stable socioeconomic conditions, greater knowledge of child-rearing practices, and established social support networks. We also found that not listening to the radio was associated with undernutrition and that watching television had a protective effect. This association could indicate a lack of access or low education level within households with a limited understanding of important information related to nutrition and health practices. Access to the radio and watching TV can provide households with valuable information and education on various topics, including nutrition and health. Education programs often disseminate public health messages, promote healthy practices, and raise awareness about nutritious food choices and childcare practices [66].

Every study has its strengths and limitations. The main strength of our study is its exclusive focus on undernutrition within Northern Africa, enabling a comprehensive analysis of the factors that contribute to this issue. By concentrating solely on this region, we gain in-depth insights into the region-specific challenges, circumstances, and contextual factors influencing undernutrition. A second strength is this study’s use of the most recent nationally representative data from the four countries of Northern Africa. This ensures that the findings are representative of the entire population, enhancing the generalisability and applicability of the results. This study’s other strengths are the inclusion of three age groups (0–59 months, 0–23 months, and 24–59 months) and the use of a population-based design, meaning that the data collected represent the entire population of the target countries. This approach increases the validity and reliability of the findings, as it avoids selection bias and allows for better estimation of prevalence and associations. Despite these strengths, this study has some acknowledged limitations. First, MICS surveys are typically cross-sectional, capturing data at a specific point in time. This limits the ability to establish temporal relationships and understand the dynamic nature of the factors contributing to undernutrition. Cross-sectional data also rely on self-reported data that may be subject to recall bias or social desirability bias. Furthermore, participants may provide answers they think are expected or may have difficulty recalling certain information accurately, leading to potential inaccuracies in the data. Lastly, this study’s findings may be influenced by confounding variables not fully accounted for in the analysis. 

This study calls for action and highlights the need for a concerted effort from governments and policymakers at national and international levels to prioritise nutrition, use a holistic approach that includes addressing undernutrition, and develop context-specific strategies based on the previously identified associated factors [4]. By taking these actions, the region can work towards meeting the global action plan on child wasting through: fostering peace, enhancing climate resilience, alleviating poverty, and guaranteeing equitable access to nutritious food; investing in healthcare, education, and social safety nets that are crucial for improving the nutritional status and overall health outcomes of vulnerable populations [4,42]; ensuring equal access to education and economic opportunities [42]; and educating and empowering women, which can help break the cycle of intergenerational undernutrition and promote healthier lifestyles and caregiving practices. An important implication of these actions is ensuring that monitoring and evaluation systems are put in place to assess the effectiveness of any intervention and to track progress over time. Regular data collection and analysis can provide valuable insights into the effectiveness of interventions, enabling policymakers to make better informed decisions and adjust strategies as needed [19].

## 5. Conclusions

In conclusion, the prevalence of undernutrition, wasting, and underweight in Northern Africa raises significant concerns. The region faces a multitude of challenges, including political instability, poverty, inequality, climate change, and epidemics, all of which contribute to food insecurity and exacerbate undernutrition. Therefore, addressing child undernutrition in Northern Africa necessitates a comprehensive and multi-sectoral approach. To effectively tackle this issue, interventions, programs, and policies should target and address undernutrition in disadvantaged families and areas. This can be achieved by improving access to healthcare and parent education on family planning and dietary intake and practice, empowering women, and promoting equitable economic opportunities. By using a holistic strategy that encompasses these key aspects, substantial progress can be made in reducing undernutrition and enhancing the overall well-being of children in the region.

## Figures and Tables

**Table 1:** Identification and categorisation of the potential variables used in this study.

Variables	Identification	Reference Category
Enabling factors		
Geographical zone	1 = Algeria; 2 = Egypt; 3 = Sudan; 4 = Tunisia	Algeria
Place of residence	1 = Urban; 2 = Rural	Living in the urban area
Underlying factors		
Household wealth index	1 = Richest; 2 = Fourth; 3 = Middle; 4 = Poor; 5 = Poorest	The richest household wealth index
Gender	1 = Male; 2 = Female	Being male
Child age	1 = 0–5; 2 = 6–11; 3 = 12–17; 4 = 18–23; 5 = 24–29; 6 = 30–35; 7 = 36–41; 8 = 42–47; 9 = 48–53; 10 = 54–59	For both 0–23 and 0–59 months: 0–5 months; for 24–59 months: 24–29
Maternal education level	1 = No schooling; 2 = Primary; 3 = Secondary and above	Secondary and above
Mother’s age	1 = 15–19 years; 2 = 20–34 years; 3 = 35–49 years	Mother aged 15–19 years
Father’s age	1 = 18–34; 2 = 35–44; 3 = 45+	Father aged 18–34 years
Mother’s age at marriage	1 = ≤18 years; 2 = >18 years	≤18 years
Marital status	1 = Married; 2 = Not married (single, divorced, or widowed)	Married
Household members	1 = 2–4; 2 = 5–10; 3 = >10	Household members 2–4 individuals
Number of children under 5	1 = 1; 2 = 2 or more	Only 1 is the number of children under 5
Birth order	1 = No previous birth; 2 = 1; 3 = 2–3; 4 = 4+	No previous birth
Maternal BMI	1 = 18.5–24.9; 2 = <18.5; 3 = 25+	Maternal BMI = 18.5–24.9 (normal)
Place of delivery	1 = Health facility; 2 = Home	Delivery in the health facility
Antenatal clinic visits	1 = 8+; 2 = 4–7; 3 = 1–3; 4 = None	Antenatal care visit 8+
Delivery assistance	1 = Skilled; 2 = Unskilled	Skilled assistance
Mode of delivery	1 = Non-caesarean; 2 = Caesarean	Non-caesarean
Cooking fuels	1 = Clean; 2 = Unclean	Clean cooking fuel
Source of drinking water	1 = Protected; 2 = Unprotected	Protected drinking water
Toilet facility	1 = Improved; 2 = Unimproved	Improved toilet facility
Listening to the radio	1 = Not at all; 2 = Yes (Almost every day; At least once a week, and Less than once a week)	Not at all
Watching TV	1 = Not at all; 2 = Yes (Almost every day; At least once a week, and Less than once a week)	Not at all
Immediate factors		
Dietary diversity	1 = 5+ foods groups; 2 = <5 foods groups	5+ foods groups
Early initiation of breastfeeding	1 = After 1 h; 2 = Withing 1 h	Breastfeeding started after 1 h
Duration of breastfeeding	1 = <12 months; 2 = >12 months	Breastfeeding continued for < 12 months
Perceived baby size	1 = Average; 2 = Small; 3 = Large	Perceive baby size average
Diarrhoea previous two weeks before each survey	1 = No; 2 = Yes	No diarrhoea in the last two weeks
Cough previous two weeks before each survey	1 = No; 2 = Yes	No cough in the last two weeks

**Table 2:** Characteristics of the study population in the four Northern African countries.

	Variables	N *	% *	%
	Geographical Zone			
Enabling factors	Algeria	16,355	43.25	43.64
	Egypt	5053	13.36	13.36
	Sudan	12,793	33.83	33.46
	Tunisia	3615	9.56	9.53
	Place of residence			
	Urban	19,651	51.96	51.85
	Rural	18,165	48.04	48.15
Underlying factors	Pooled household wealth index			
	Poorest	7242	19.15	19.80
	Poor	6903	18.25	19.64
	Middle	7520	19.89	20.18
	Fourth	7938	20.99	20.39
	Richest	8214	21.72	20.00
	Gender			
	Male	19,314	51.07	51.15
	Female	18,502	48.93	48.85
	Child age			
	0–5	3610	9.546	9.68
	6–11	3782	10	9.95
	12–17	4035	10.67	10.41
	18–23	3534	9.35	9.28
	24–29	3767	9.96	10.05
	30–35	3628	9.59	9.61
	36–41	4271	11.3	11.28
	42–47	3826	10.12	10.18
	48–53	3809	10.07	10.18
	54–59	3554	9.40	9.38
	Mother’s age			
	15–19 years	1619	4.37	4.90
	20–34 years	23,756	64.09	63.81
	35–49 years	11,690	31.54	31.29
	Father’s age			
	18–34 years	6596	19.41	19.39
	35–44 years	19,229	56.59	56.32
	45+ years	8153	23.99	24.29
	Mother’s age at marriage			
	<18 years	88,649	24.75	25.38
	>18 years	26,289	75.25	74.62
	Marital status			
	Married	34,295	96.02	96.01
	Not married	1422	3.981	3.99
	Maternal education level			
	No schooling	11,454	32.15	33.90
	Primary	11,674	32.77	31.63
	Secondary and above	12,501	35.09	34.48
	Maternal BMI			
	18.5–24.9	5366	14.19	13.66
	<18.5	32,098	84.88	85.34
	25+	350	0.925	1.00
	Number of household members			
	2–4	10,260	27.13	26.83
	5–10	24,992	66.09	66.35
	>10	2564	6.78	6.82
	Number of children under 5			
	1	17,300	45.75	46.05
	2 or more	20,516	54.25	53.95
	Birth order			
	No previous birth	7644	20.21	19.33
	1	14,649	38.74	39.43
	2–3	11,446	30.27	30.80
	4+	4077	10.78	10.44
	Place of delivery			
	Health facility	9795	71.79	70.53
	Home	3850	28.21	29.47
	Antenatal clinic visits			
	8+	2236	14.94	14.65
	4–7	6881	45.99	44.31
	1–3	3172	21.2	22.41
	None	2672	17.86	18.63
	Delivery assistance			
	Skilled	12,386	82.79	81.45
	Unskilled	2575	17.21	18.55
	Mode of delivery			
	Non-caesarean	6444	65.68	66.75
	Caesarean	3367	34.32	33.25
	Cooking fuels			
	Clean	79,824	78.87	75.60
	Unclean	7988	21.13	24.40
	Source of drinking water			
	Protected	28,418	75.2	74.20
	Unprotected	9372	24.8	25.80
	Toilet facility			
	Improved	21,009	55.62	55.22
	Unimproved	16,766	44.38	44.78
	Listening to the radio			
	Not at all	27,619	73.9	73.26
	Yes ^	9756	26.1	26.74
	Watching TV			
	Not at all	9205	24.62	26.09
	Yes ^	28,182	75.38	73.91
Immediate factors	Dietary diversity			
	5+ foods	4574	30.58	29.62
	<5 foods	10,386	69.42	70.38
	Early initiation of breastfeeding			
	After 1 h	7221	48.27	47.33
	Within 1 h	7739	51.73	52.67
	Perceived baby size			
	Average	8423	62.29	63.09
	Small	3208	23.72	23.08
	Large	1891	13.98	13.82
	Duration of breastfeeding			
	<12 months	6910	46.19	46.77
	>12 months	8050	53.81	53.23
	Diarrhoea previous two weeks			
	No	31,509	83.65	84.77
	Yes	6158	16.35	15.23
	Cough the previous two weeks			
	No	26,992	71.48	71.97
	Yes	10,767	28.52	28.03

* = Weighted number and percentage; ^ = Almost every day; At least once a week and less than once a week.

**Table 3:** Factors associated with wasting among children under five in Northern Africa: unadjusted and adjusted odds ratios (ORs).

Variables	Wasted Children 0–23 Months				Wasted Children 24–59 Months				Wasted Children 0–59 Months			
	Unadjusted Odds Ratio (OR)		Adjusted Odds Ratio (AOR)		Unadjusted Odds Ratio (OR)		Adjusted Odds Ratio (AOR)		Unadjusted Odds Ratio (OR)		Adjusted Odds Ratio (AOR)	
	OR (95% CI)	*p*-Value	aOR (95% CI)	*p*-Value	OR (95% CI)	*p*-Value	aOR (95% CI)	*p*-Value	OR (95% CI)	*p*-Value	aOR (95% CI)	*p*-Value
Enabling factors												
Geographical zone												
Algeria	1.00		1.00		1.00		1.00		1.00		1.00	
Egypt	0.89 (0.58, 1.35)	0.568	0.68 (0.42, 1.10)	0.115	0.94 (0.58, 1.52)	0.787	1.00 (0.59, 1.69)	0.995	0.95 (0.66, 1.37)	0.791	0.94 (0.65, 1.37)	0.762
Sudan	4.41 (3.27, 5.93)	<0.001	3.25 (2.21, 4.78)	<0.001	10.88 (8.56, 13.83)	<0.001	14.58 (10.55, 20.16)	<0.001	6.65 (5.29, 8.37)	<0.001	6.16 (4.69, 8.12)	<0.001
Tunisia	0.61 (0.38, 0.96)	0.034	0.66 (0.42, 1.02)	0.063	0.86 (0.56, 1.31)	0.472	0.93 (0.61, 1.42)	0.735	0.68 (0.49, 0.95)	0.025	0.69 (0.49, 0.97)	0.032
Underlying factors												
Pooled household wealth index												
Richest	-	-	-	-	1.00		1.00		1.00		1.00	
Rich	-	-	-	-	0.95 (0.74, 1.22)	0.691	1.43 (1.07, 1.92)	0.016	1.35 (1.06, 1.71)	0.015	1.29 (1.01, 1.63)	0.037
Middle	-	-	-	-	0.67 (0.51, 0.89)	0.005	0.99 (1.73, 1.33)	0.924	0.75 (0.59, 0.96)	0.024	1.01 (0.79, 1.29)	0.951
Poor	-	-	-	-	1.44 (1.08, 1.92)	0.013	1.41 (1.05, 1.88)	0.021	0.85 (0.69, 1.05)	0.131	1.10 (0.88, 1.38)	0.418
Poorest	-	-	-	-	1.63 (1.24, 2.13)	<0.001	1.85 (1.42, 2.40)	<0.001	1.24 (0.97, 1.59)	0.082	1.39 (1.09, 1.79)	0.009
Gender												
Male	-	-	-	-	1.00		1.00		1.00		1.00	
Female	-	-	-	-	0.79 (0.98, 0.92)	0.003	0.77 (0.66, 0.92)	0.001	0.88 (0.78, 1.00)	0.049	0.88 (0.78, 1.00)	0.044
Child age												
0–5	-	-	-	-	-	-	-	-	1.00		1.00	
6–11	-	-	-	-	-	-	-	-	1.15 (0.88, 1.51)	0.314	1.12 (0.82, 1.52)	0.477
12–17	-	-	-	-	-	-	-	-	1.08 (0.88, 1.33)	0.442	1.10 (0.87, 1.39)	0.415
18–23	-	-	-	-	-	-	-	-	0.80 (0.62, 1.03)	0.084	0.87 (0.66, 1.15)	0.322
24–29	-	-	-	-	1.00		1.00		0.88 (0.70, 1.10)	0.25	0.85 (0.67, 1.09)	0.198
30–35	-	-	-	-	0.69 (0.55, 0.87)	0.001	0.79 (0.62, 1.01)	0.061	0.60 (0.48, 0.76)	<0.001	0.67 (0.51, 0.86)	0.002
36–41	-	-	-	-	0.66 (0.50, 0.87)	0.004	0.70 (0.50, 0.98)	0.038	0.58 (0.44, 0.76)	<0.001	0.51 (0.39, 0.67)	<0.001
42–47	-	-	-	-	0.76 (0.60, 0.96)	0.023	0.76 (0.56, 1.03)	0.075	0.66 (0.52, 0.85)	0.001	0.62 (0.48, 0.80)	<0.001
48–53	-	-	-	-	0.74 (0.58, 0.94)	0.014	0.81 (0.60, 1.09)	0.157	0.65 (0.52, 0.81)	<0.001	0.62 (0.49, 0.79)	<0.001
54–59	-	-	-	-	0.72 (0.56, 0.94)	0.014	1.01 (0.75, 1.37)	0.944	0.63 (0.48, 0.84)	0.001	0.68 (0.52, 0.90)	0.006
Father’s age												
18–34	-	-	-	-	1.00		1.00		-	-	-	-
35–44	-	-	-	-	0.61 (0.51, 0.74)	<0.001	1.11 (0.92, 1.35)	0.278	-	-	-	-
45+	-	-	-	-	0.70 (0.57, 0.86)	0.001	0.79 (0.64, 0.98)	0.031	-	-	-	-
Birth order												
No previous brith	1.00		1.00									
1	2.27 (1.76, 2.92)	<0.001	1.43 (1.06, 1.93)	0.02	-	-	-	-	-	-	-	-
2–3	2.88 (2.13, 3.91)	<0.001	1.58 (1.03, 2.42)	0.038	-	-	-	-	-	-	-	-
4+	4.41 (3.26, 5.96)	<0.001	1.64 (1.13, 2.38)	0.01	-	-	-	-	-	-	-	-
Immediate factors												
Dietary diversity												
5+ food groups	1.00		1.00		-	-	-	-	1.00		1.00	
<5 food groups	2.07 (1.67, 2.56)	<0.001	1.51 (1.20, 1.90)	<0.001	-	-	-	-	1.66 (1.41, 1.97)	<0.001	1.23 (1.00, 1.52)	0.049
Diarrhoea in the previous two weeks												
No	-	-	-	-	-	-	-	-	1.00		1.00	
Yes	-	-	-	-	-	-	-	-	2.27 (1.94, 2.64)	<0.001	1.22 (1.02, 1.46)	0.024

**Table 4:** Factors associated with underweight among children under five in Northern Africa: unadjusted and adjusted odds ratios (ORs).

Variables	Underweight Children 0–30 Months				Underweight Children 24–59 Months				Underweight Children 0–59 Months			
	Unadjusted Odds Ratio (OR)		Adjusted Odds Ratio (AOR)		Unadjusted Odds Ratio (OR)		Adjusted Odds Ratio (AOR)		Unadjusted Odds Ratio (OR)		Adjusted Odds Ratio (AOR)	
	OR (95% CI)	*p*-Value	aOR(95% CI)	*p*-Value	OR (95% CI)	*p*-Value	aOR(95% CI)	*p*-Value	OR (95% CI)	*p*-Value	aOR(95% CI)	*p*-Value
Enabling factors												
Geographical zone												
Algeria	1.00		1.00		1.00		1.00		1.00		1.00	
Egypt	2.35 (1.44,3.84)	0.001	1.97 (1.18, 3.28)	0.010	1.70 (1.15, 2.52)	0.008	2.17 (1.46, 3.22)	<0.001	2.10 (1.54, 2.86)	<0.001	2.47 (1.79, 3.40)	<0.001
Sudan	9.27 (7.34, 11.70)	<0.001	5.9 (4.48, 7.78)	<0.001	22.83 (18.41, 28.31)	<0.001	20.56 (16.13, 26.20)	<0.001	15.67 (13.25, 18.53)	<0.001	9.17 (7.18, 11.72)	<0.001
Tunisia	0.74 (0.47, 1.14)	0.171	0.75 (0.48, 1.18)	0.212	0.45 (0.27, 0.74)	0.002	0.45 (0.27, 0.77)	0.003	0.58 (0.41, 0.81)	0.001	0.60 (0.42, 0.86)	0.005
Place of residence												
Urban	1.00		1.00		1.00		1.00		1.00		1.00	
Rural	2.31 (1.88, 2.85)	<0.001	1.24 (1.00, 1.53)	0.047	3.79 (3.14, 4.59)	<0.001	1.58 (1.31, 1.89)	<0.001	3.12 (2.65, 3.69)	<0.001	1.43 (1.24, 1.64)	<0.001
Underlying factors												
Pooled household wealth index												
Richest	1.00		1.00		1.00		1.00		-	-	-	-
Rich	0.89 (0.70, 1.15)	0.374	1.12 (0.84, 1.48)	0.441	1.11 (0.89, 1.37)	0.344	1.36 (1.07, 1.71)	0.011	-	-	-	-
Middle	1.1 (0.83, 1.46)	0.502	1.33 (0.99, 1.80)	0.058	1.04 (0.83, 1.29)	0.75	1.21 (0.95, 1.55)	0.121	-	-	-	-
Poor	1.45 (1.14, 1.85)	0.002	1.37 (1.03, 1.81)	0.030	1.82 (1.47, 2.25)	<0.001	1.15 (0.87, 1.51)	0.325	-	-	-	-
Poorest	1.17 (0.90, 1.50)	0.239	1.09 (0.80, 1.48)	0.595	1.43 (1.14, 1.80)	0.002	0.99 (0.73, 1.36)	0.965	-	-	-	-
Gender												
Male	1.00		1.00		-	-	-	-	1.00		1.00	
Female	0.87 (0.75, 1.0)	0.045	0.79 (0.68, 0.92)	0.002	-	-	-	-	0.90 (0.84, 0.98)	0.011	0.88 (0.80, 0.96)	0.005
Child age												
0–5	1.00		1.00		-	-	-	-	1.00		1.00	
6–11	1.22 (1.00, 1.48)	0.048	1.23 (0.98, 1.54)	0.071	-	-	-	-	1.22 (1.00, 1.48)	0.048	1.18 (0.92, 1.52)	0.195
12–17	1.29 (1.05, 1.58)	0.014	0.93 (0.62, 1.39)	0.724	-	-	-	-	1.29 (1.05,1.58)	0.014	1.19 (0.92,1.56)	0.188
18–23	1.34 (1.10, 1.63)	0.003	1.04 (0.66, 1.62)	0.870	-	-	-	-	1.34 (1.10, 1.63)	0.003	1.39 (1.06, 1.81)	0.016
24–29	-	-	-	-	1.00		1.00		1.48 (1.16, 1.89)	0.002	1.32 (0.98, 1.78)	0.071
30–35	-	-	-	-	0.99 (0.82, 1.20)	0.94	1.28 (1.03, 1.58)	0.025	1.47 (1.23, 1.76)	<0.001	1.62 (1.24, 2.11)	<0.001
36–41	-	-	-	-	0.86 (0.11, 1.03)	0.109	0.80 (0.66, 0.97)	0.025	1.28 (1.02, 1.59)	0.032	0.93 (0.69, 1.24)	0.608
42–47	-	-	-	-	1.09 (0.89, 1.32)	0.41	1.13 (0.92, 1.39)	0.242	1.61 (1.31, 1.98)	<0.001	1.27 (0.97, 1.66)	0.084
48–53	-	-	-	-	0.78 (0.63, 0.97)	0.022	0.82 (0.65, 1.03)	0.083	1.16 (0.94, 1.42)	0.171	0.92 (0.70, 1.21)	0.555
54–59	-	-	-	-	0.86 (0.70, 1.04)	0.125	1.09 (0.88, 1.35)	0.442	1.27 (0.98, 1.64)	0.072	1.18 (0.90, 1.55)	0.239
Maternal Education level												
No schooling	-	-	-	-	1.00		1.00		1.00		1.00	
Primary	-	-	-	-	2.49 (2.11, 2.93)	<0.001	1.62 (1.33, 1.98)	<0.001	2.10 (1.82, 2.43)	<0.001	1.37 (1.18, 1.59)	<0.001
Secondary and above	-	-	-	-	3.73 (3.08, 4.51)	<0.001	1.94 (1.52, 2.47)	<0.001	2.98 (2.52, 3.51)	<0.001	1.58 (1.34, 1.86)	<0.001
Maternal BMI												
19–25	-	-	-	-	-	-	-	-	1.00		1.00	
<=18.5	-	-	-	-	-	-	-	-	15.28 (10.90, 21.41)	<0.001	8.31 (5.71, 12.09)	<0.001
25+	-	-	-	-	-	-	-	-	3.05 (1.66, 2.60)	<0.001	2.06 (1.05, 4.04)	0.035
Listening to the radio												
Not at all	-	-	-	-	1.00		1.00		1.00		1.00	
Yes ^	-	-	-	-	1.08 (0.94, 1.24)	0.265	0.79 (0.68, 0.92)	0.003	1.10 (0.99, 1.23)	0.085	0.81 (0.73, 0.94)	0.004
Watching TV												
Not at all	-	-	-	-	-	-	-	-	1.00		1.00	
Yes ^	-	-	-	-	-	-	-	-	0.17 (0.15, 1.95)	<0.001	0.85 (0.73, 0.99)	0.033
Immediate factors												
Dietary diversity												
5+ food groups	1.00		1.00		-	-	-	-	1.00		1.00	
<5 food groups	2.29 (1.87, 2.80)	<0.001	1.58 (1.27, 1.98)	<0.001	-	-	-	-	2.08 (1.80, 2.41)	<0.001	1.20 (1.02, 1.41)	0.025
Duration of breastfeeding												
≤12 months	1.00		1.00		-	-	-	-				
>12 months	1.07 (0.94, 1.22)	0.283	1.55 (1.04, 2.31)	0.030	-	-	-	-				
Perceived baby size												
Normal	1.00		1.00		-	-	-	-	-	-	-	-
Small	2.37 (0.03, 2.78)	<0.001	1.60 (1.36, 1.88)	<0.001	-	-	-	-	-	-	-	-
Large	0.86 (0.69, 1.08)	0.187	0.79 (0.63, 0.98)	0.034	-	-	-	-	-	-	-	-
Diarrhoea in the previous two weeks												
No	1.00		1.00		1.00		1.00		1.00		1.00	
Yes	2.26 (1.91, 2.68)	<0.001	1.22 (1.03, 1.45)	0.021	3.33 (0.86, 3.86)	<0.001	1.38 (1.18, 1.61)	<0.001	2.70 (2.37, 3.06)	<0.001	1.35 (1.19, 1.53)	<0.001

^ = Almost every day; At least once a week and less than once a week.

## Data Availability

Data are available online at https://mics.unicef.org/surveys (accessed on 21 June 2023).

## Supplementary Materials

The following supporting information can be downloaded at: https://www.mdpi.com/article/10.3390/nu15143207/s1. Table S1. Prevalence and 95% confidence intervals (CIs) of wasting and underweight among children (0–23, 24–59, and 0–59 months) in four Northern African countries, N = 37,816.
