# Supplementary material for: Wasting and Underweight in Northern African Children: Findings from Multiple-Indicator Cluster Surveys, 2014–2018
Source: Nutrients. 2023 Jul 19;15(14):3207. doi: 10.3390/nu15143207 (PMC10384034; doi:10.3390/nu15143207)
Supplement: Supplementary file 1 [file nutrients-15-03207-s001.zip › nutrients-2489697-supplementary.pdf]

Table S1. Prevalence and 95% confidence intervals (CIs) of wasting and underweight among children (0-23, 24-59 and 0-59 months) in four Northern African countries, N= 37816.

Variables	Wasted children (0-23 months)	Wasted children (24-59 months)	Wasted children (0-59 months)	Children underweight (0-23 months)	Children underweight (24-59 months)	Children underweight (0-59 months)
	% (95% CI)	% (95% CI)	% (95% CI)	% (95% CI)	% (95% CI)	% (95% CI)
**Geographical zone**						
Algeria	4.68 [4.00,5.48)	4.7 [4.00, 5.48]	1.6 [1.306, 1.897]	3.41 [2.897, 4.00]	2.1 [1.79, 2.48]	2.6 [2.33, 2.93]
Egypt	4.17 [3.31,5.23]	4.2 [3.31, 5.23]	1.5 [1.069, 2.034]	7.65 [6.46, 9.03]	3.5 [2.82, 4.43]	5.3 [4.66, 6.11]
Sudan	17.79 [16.42, 19.26]	17.8 [16.42, 19.26]	14.8 [13.78, 15.93]	24.63 [23.11, 26.21]	33.0 [31.6, 34.38]	29.6 [28.57, 30.66]
Tunisia	2.90 [2.06, 4.08]	2.9 [2.06, 4.08]	1.4 [0.95, 1.92]	2.53 [1.78, 3.59]	1.0 [0.62, 1.45]	1.5 [1.16, 2.00]
**Place of residence**						
Urban	6.7 [5.6,8.0]	3.4 (2.9, 3.9)	4.7 [4.1,5.4]	7.3 [6.2,8.6]	6.1 [5.4,6.9]	6.6 [5.9,7.3]
Rural	11.3 [10.1,12.5]	8.9 (7.9, 10.0)	9.8 [9.0,10.8]	15.5 [14.1,17.0]	19.8 [17.9,21.7]	18.0 [16.5,19.6]
**Household wealth Index**						
Poorest	8.6 [7.2,10.2]	8.5 [7.2,10.0]	8.5 [7.4,9.8]	11.8 [10.2,13.7]	14.2 [12.4,16.1]	13.3 [11.8,14.9]
Poor	11.5 [9.9,13.4]	7.6 [6.3,9.2]	9.2 [8.1,10.4]	14.3 [12.6,16.2]	17.3 [15.6,19.2]	16.1 [14.7,17.6]
Middle	8.0 [6.5,9.8]	3.7 [3.0,4.5]	5.4 [4.6,6.2]	11.2 [9.4,13.4]	10.7 [9.2,12.3]	10.9 [9.6,12.3]
Fourth	7.2 [5.9,8.7]	5.1 [4.2,6.3]	6.0 [5.1,7.1]	9.3 [7.6,11.3]	11.3 [9.0,14.2]	10.5 [8.6,12.7]
Richest	9.4 [7.2,12.1]	5.4 [4.5,6.5]	7.0 [5.8,8.3]	10.3 [8.7,12.2]	10.3 [8.9,12.0]	10.3 [9.0,11.8]
**Gender**						
Boy	8.9 [7.7,10.2]	6.7 [6.0,7.5]	7.6 [6.8,8.3]	12.0 [10.8,13.3]	13 [11.9,14.3]	12.6 [11.6,13.7]
Girl	8.9 [8.0,9.8]	5.4 [4.7,6.1]	6.7 [6.2,7.4]	10.6 [9.5,11.7]	12.2 [11.1,13.4]	11.6 [10.7,12.5]
**Child age**						
0-5	8.8 [7.7,10.1]		8.8 [7.7,10.1]	9.5 [8.3,10.9]		9.5 [8.3,10.9]
6-11	10.0 [8.1,12.3]		10.0 [8.1,12.3]	11.4 [9.9,13.1]		11.4 [9.9,13.1]
12-17	9.5 [8.2,10.9]		9.5 [8.2,10.9]	12.0 [10.5,13.6]		12.0 [10.5,13.6]
18-23	7.2 [5.9,8.6]		7.2 [5.9,8.6]	12.4 [10.9,14.0]		12.4 [10.9,14.0]
24-29	-	7.8 [6.6,9.1]	7.8 [6.6,9.1]	-	13.5 [11.6,15.7]	13.5 [11.6,15.7]
30-35	-	5.5 [4.6,6.6]	5.5 [4.6,6.6]	-	13.4 [12.0,14.9]	13.4 [12.0,14.9]
36-41	-	5.3 [4.3,6.5]	5.3 [4.3,6.5]		11.8 [10.3,13.6]	11.8 [10.3,13.6]
42-47	-	6 [5.0,7.2]	6.0 [5.0,7.2]	-	14.5 [12.9,16.3]	14.5 [12.9,16.3]
48-53	-	5.9 [5.0,6.9]	5.9 [5.0,6.9]	-	10.9 [9.6,12.2]	10.9 [9.6,12.2]
54-59	-	5.7 [4.6,7.2]	5.7 [4.6,7.2]	-	11.8 [9.8,14.1]	11.8 [9.8,14.1]
**Mother's age**						
15-19 years	8.8 [6.2,12.2]	4.9 [3.1,7.6]	6.7 [4.8,9.2]	13.7 [10.5,17.7]	8.2 [6.0,11.1]	10.8 [8.7,13.4]
20-34 years	9.2 [8.3,10.1]	6.4 [5.7,7.2]	7.6 [7.0,8.3]	11.5 [10.5,12.6]	13.8 [12.6,15.0]	12.8 [11.9,13.8]
35-49years	7.8 [6.8,9.0]	5.3 [4.6,6.1]	6.1 [5.5,6.8]	10.5 [9.2,11.9]	11 [9.6,12.5]	10.8 [9.7,12.0]
**Father’s age**						
18-34	9.8 [8.5,11.4]	8.7 [7.4,10.3]	9.3 [8.2,10.5]	15.4 [13.4,17.7]	19.7 [17.6,21.9]	17.6 [15.8,19.4]
35-44	8.0 [7.1,9.1]	5.6 [4.9,6.3]	6.5 [6.0,7.2]	9.4 [8.5,10.5]	9.9 [9.0,11.0]	9.7 [8.9,10.6]
45+	10.8 [9.2,12.7]	6.3 [5.5,7.3]	7.8 [6.9,8.8]	14.5 [12.8,16.3]	16.3 [14.5,18.3]	15.7 [14.1,17.4]
**Mother’s age at marriage**						
< 18 years	15.2 [13.3,17.2]	11.4 [10.0,12.9]	12.9 [11.7,14.2]	20.8 [18.9,23.0]	27.1 [24.9,29.5]	24.5 [22.7,26.4]
>18 years	6.9 [6.3,7.7]	4.6 [4.1,5.1]	5.5 [5.1,6.0]	8.7 [7.8,9.6]	8.7 [7.8,9.6]	8.7 [8.0,9.4]
**Marital status**						
Married	9.0 [8.2,9.8]	6.2 [5.6,6.9]	7.3 [6.8,7.9]	11.8 [10.8,12.8]	13.1 [12.1,14.3]	12.6 [11.7,13.5]
Not married	12.2 [5.6,24.7]	8.2 [6.1,11.1]	9.4 [6.6,13.3]	10.1 [6.7,14.9]	16.1 [13.5,19.2]	14.4 [12.2,16.9]

**Maternal education level**						
No schooling	11.3 [10.0,12.7]	3.9 [3.3,4.7]	9.5 [8.7,10.4]	15.3 [13.8,16.9]	6.2 [5.4,7.0]	18.0 [16.4,19.7]
Primary	9.5 [8.2,11.0]	6.5 [5.6,7.6]	7.7 [6.9,8.6]	12.6 [11.1,14.1]	14 [12.6,15.6]	13.4 [12.2,14.7]
Secondary and above	7.1 [5.7,8.7]	8.5 [7.5,9.6]	5.3 [4.6,6.1]	7.8 [6.7,9.1]	19.7 [17.7,21.8]	6.9 [6.1,7.7]
**Maternal BMI**						
19-25	0	0	0	0.8 [0.5,1.3]	1.4 [0.9,2.2]	1.1 [0.8,1.5]
<=18.5	11.3 [10.3,12.4]	6.8 [6.1, 7.4]	8.4 [7.8,9.1]	14.0 [13.0,15.2]	13.9 [12.8,15.1]	14.0 [13.0,15.0]
25+	0	0	0	3.8 [1.6,8.7]	2.7 [1.2,6.0]	3.1 [1.8,5.6]
**Household members**						
2-4	7.3 [6.3,8.5]	4.5 [3.8,5.4]	5.8 [5.1,6.5]	10.2 [8.8,11.7]	8.2 [7.3,9.3]	9.1 [8.2,10.1]
5-10	9.2 [8.4,10.0]	6.5 [5.8,7.2]	7.5 [6.9,8.1]	11.4 [10.4,12.5]	13.8 [12.7,15.0]	12.9 [12.0,13.9]
>10	13.1 [8.1,20.4]	6.9 [5.2,9.1]	9.5 [6.9,13.0]	14.9 [11.6,18.8]	17.2 [13.7,21.4]	16.2 [13.6,19.3]
**Number of children under 5**						
1	7.3 [6.1,8.7]	4.5 [4.0,5.2]	5.6 [5.0,6.3]	8.5 [7.6,9.6]	8.9 [8.1,9.7]	8.7 [8.1,9.4]
2 or more	10.2 [9.2,11.2]	7.3 [6.6,8.2]	8.5 [7.8,9.2]	13.5 [12.3,14.7]	15.9 [14.5,17.5]	14.9 [13.8,16.2]
**Birth order**						
Non previous	4.1 [3.3,5.0]	1.6 [1.2,2.2]	2.8 [2.4,3.4]	4.4 [3.6,5.4]	2 [1.5,2.8]	3.2 [2.7,3.8]
1	8.8 [7.8,9.9]	4.9 [4.3,5.6]	6.4 [5.8,7.0]	11.5 [10.2,13.0]	10.4 [9.4,11.5]	10.8 [9.9,11.8]
2-3	11.0 [9.1,13.2]	7.7 [6.8,8.8]	8.9 [7.8,10.2]	13.1 [11.6,14.7]	16.1 [14.7,17.7]	15.0 [13.8,16.3]
4+	16 [13.4,18.9]	11.6 [9.8,13.5]	13.1 [11.7,14.7]	23.2 [20.1,26.6]	26 [22.8,29.5]	25.0 [22.5,27.6]
**Place of delivery**						
Health facility	5.9 [5.1,6.7]		-	6.8 [6.0,7.7]		-
Home	17.2 [15.6,18.9]		-	24.4 [22.7,26.3]		-
**Antenatal clinic visits**						
8+	5.6 [4.4,7.0]		-	7.2 [5.8,9.0]		-
4-7	8.0 [7.0,9.0]		-	10.5 [9.4,11.7]		-
1-3	10.8 [9.4,12.4]		-	14.1 [12.5,16.0]		-
None	11.8 [9.5,14.5]		-	13.2 [11.5,15.2]		-
**Delivery assistance**						
Skilled	8.4 [7.6,9.3]		-	10.7 [9.8,11.8]		-
Unskilled	11.2 [8.9,13.9]		-	14.0 [12.2,15.9]		-
**Mode of delivery**						
Non-caesarean	5.6 [4.8,6.5]		-	6.6 [5.7,7.6]		-
Caesarean	6.5 [5.4,7.9]		-	7.4 [6.0,9.1]		-
**Cooking fuels**						
Clean	6.3 [5.4,7.3]	3.2 [2.8,3.7]	4.4 [3.9,5.0]	7.2 [6.3,8.3]	6.6 [5.6,7.7]	6.8 [6.0,7.8]
Un clean	18.7 [16.9,20.8]	16.4 [14.6,18.3]	17.3 [16.0,18.7]	26 [23.9,28.1]	34.9 [32.7,37.2]	31.3 [29.5,33.2]
**Source of drinking water**						
Protected	9.0 [8.0,10.1]	5.8 [5.1,6.5]	7.0 [6.4,7.7]	11.5 [10.4,12.7]	12.6 [11.4,14.0]	12.2 [11.1,13.3]
Unprotected	8.6 [7.4,10.0]	6.8 [5.8,7.9]	7.5 [6.5,8.5]	10.6 [9.2,12.2]	12.6 [11.2,14.3]	11.9 [10.6,13.2]
**Toilet facility**						
Improved	7.2 [6.1,8.4]	3.8 [3.4,4.4]	5.2 [4.6,5.8]	8.9 [7.8,10.2]	8 [7.1,9.1]	8.4 [7.6,9.3]
Unimproved	11.1 [10.0,12.4]	8.7 [7.6,9.8]	9.6 [8.8,10.6]	14.3 [12.9,15.8]	18.3 [16.6,20.1]	16.7 [15.3,18.2]
**Listening to the radio**						
Not at all	8.7 [7.8,9.6]	5.8 [5.2,6.4]	6.9 [6.4,7.5]	11.0[10.0,12.2]	12.5 [11.4,13.8]	11.9 [11.0,13.0]
Yes	9.3 [8.0,10.7]	6.9 [5.9,8.0]	7.8 [7.0,8.8]	12.4 [11.2,13.8]	13.4 [12.1,14.8]	13.0 [11.9,14.2]
**Watching TV**						

Not at all	17.5 [15.8,19.3]	14.7 [13.0,16.4]	15.8 [14.5,17.1]	23.8[21.8,25.8]	32.8 [30.2,35.5]	29.2 [27.2,31.2]
Yes	6.0 [5.3,6.8]	3.3 [2.9,3.7]	4.4 [3.9,4.8]	7.2 [6.4,8.1]	6.1 [5.5,6.9]	6.6 [6.0,7.2]
**Dietary diversity**						
5+ foods	5.3 [4.5,6.3]	3.9 [3.1,4.9]	4.8 [4.1,5.5]	6.4 [5.3,7.6]	7.8 [6.6,9.2]	6.9 [6.1,7.8]
<5 foods	10.4 [9.4,11.6]	6.3 [5.7,7.0]	7.7 [7.1,8.4]	13.4 [12.3,14.6]	13.3 [12.2,14.5]	13.4 [12.4,14.4]
**Early initiation of breast feeding**						
After 1 hr	6.6 [5.7,7.8]		-	7.8 [6.8,8.9]		-
Withing 1 hr	11.0 [10.0,12.1]		-	14.6 [13.4,15.8]		-
**Duration of breast feeding**						
< 12 months	9.3 [8.4, 10.3]			10.9 [9.9, 12.1]		
>12 months	8.6 [7.5, 9.8]			11.6 [10.5, 12.9]		
**Size of baby**						
Average	8.0 [7.1,8.9]	5.8 [4.9,7.0]	7.1 [6.3,7.9]	9.5 [8.5,10.6]	10.9 [9.9,12.0]	10.9 [9.9,12.0]
Small	12.7 [11.2,14.4]	8.6 [7.0,10.4]	11.0 [9.8,12.3]	20.0 [17.9,22.2]	20.3 [18.4,22.3]	20.3 [18.4,22.3]
Large	7.4 [6.0,9.0]	6.9 [5.3,9.1]	7.2 [6.1,8.6]	8.3 [6.9,10.0]	10.6 [9.2,12.2]	10.6 [9.2,12.2]
**Diarrhoea previous two weeks**						
No	7.6 [6.9,8.3]	5.2 [4.6,5.8]	6.0 [5.5,6.6]	9.2 [8.4,10.1]	10.4 [9.5,11.4]	10.0 [9.2,10.8]
Yes	13.4 [11.0,16.1]	12 [10.5,13.6]	12.7 [11.2,14.4]	18.7 [16.5,21.1]	27.9 [25.3,30.6]	23.0 [21.0,25.1]
**Cough previous two weeks**						
No	8.5 [7.7,9.4]	5.7 [5.0,6.4]	6.8 [6.2,7.4]	11.0 [10.1,12.0]	12 [10.9,13.2]	11.6 [10.7,12.6]
Yes	9.8 [7.9,12.1]	6.9 [6.1,7.8]	8.0 [7.0,9.1]	12.0 [10.5,13.8]	14.2 [13.0,15.5]	13.3 [12.2,14.5]
